# Glycemic control by umbilical cord-derived mesenchymal stem cells promotes effects of fasting-mimicking diet on type 2 diabetic mice

**DOI:** 10.1186/s13287-021-02467-7

**Published:** 2021-07-13

**Authors:** Na Zhao, Ying-Feng Gao, Lei Bao, Jing Lei, Huan-Xiao An, Feng-Xing Pu, Rui-Ping Cheng, Ji Chen, Hua Ni, Bing-Dong Sui, Fan-Pu Ji, Cheng-Hu Hu

**Affiliations:** 1 Institute for Stem Cell & Regenerative Medicine, The Second Affiliated Hospital of Xi’an Jiaotong University, Xi’an, Shaanxi People’s Republic of China; 2 National & Local Joint Engineering Research Center of Biodiagnosis and Biotherapy, The Second Affiliated Hospital of Xi’an Jiaotong University, Xi’an, Shaanxi People’s Republic of China; 3 Xi’an Institute of Tissue Engineering and Regenerative Medicine, Xi’an, Shaanxi People’s Republic of China; 4 Department of Obstetrics and Gynecology, Xi’an No. 4 Hospital, Affiliated Guangren Hospital, School of Medicine, Xi’an Jiaotong University, Xi’an, Shaanxi People’s Republic of China; 5 State Key Laboratory of Military Stomatology & National Clinical Research Center for Oral Diseases & Shaanxi International Joint Research Center for Oral Diseases, Center for Tissue Engineering, School of Stomatology, The Fourth Military Medical University, Xi’an, Shaanxi People’s Republic of China; 6 Department of Infectious Diseases, The Second Affiliated Hospital of Xi’an Jiaotong University, Xi’an, Shaanxi People’s Republic of China; 7 Key Laboratory of Environment and Genes Related to Diseases, Xi’an Jiaotong University, Ministry of Education of China, Xi’an, Shaanxi People’s Republic of China

**Keywords:** Fasting-mimicking diet, Glycometabolism, Human umbilical cord-derived mesenchymal stem cells, Inflammatory cytokines, Lipometabolism, Type 2 diabetes

## Abstract

**Background:**

Hepatic steatosis is a big hurdle to treat type 2 diabetes (T2D). Fasting-mimicking diet (FMD) has been shown to be an effective intervention in dyslipidemia of T2D. However, fasting may impair the normal glucose metabolism. Human umbilical cord-derived mesenchymal stem cell (UC-MSC) transplantation has been discovered to regulate immune reactions and reduce hyperglycemia in diabetes. However, the effect of UC-MSCs on improving the lipid metabolism disorder is not quite satisfactory. We have investigated the efficacy comparison and interaction between FMD and UC-MSC infusion, aiming to establish effective T2D therapies and explore its mechanism.

**Methods:**

C57/BL6 mice were fed with high-fat diet (HFD) to induce a diet-induced obese (DIO) mouse model. Leptin receptor-deficient (db/db) mice were used for follow-up experiments. DIO or db/db mice were divided into 4 groups: phosphate buffer saline (PBS), UC-MSCs, FMD, and UC-MSCs + FMD. At the end of the study period, mice were fasted and sacrificed, with the measurement of physiological and biochemical indexes. In addition, the fresh liver, skin, and white adipose tissue were analyzed by histology.

**Results:**

FMD restored the lipid metabolism in DIO mice, whereas its capacity to rescue hyperglycemia was uncertain. Infusion of UC-MSCs was effective in T2D glycemic control but the impact on dyslipidemia was insufficient. Furthermore, both the glucose and the lipid alterations of DIO and db/db mice recovered after UC-MSCs combined with FMD. It was proved that UC-MSCs promoted FMD effects on ameliorating hyperglycemia and restoring the lipid metabolism in T2D mice, while FMD had little promotion effect on UC-MSCs. Mechanistically, we discovered that UC-MSC infusion significantly modulated systematic inflammatory microenvironment, which contributed to concerted actions with FMD.

**Conclusions:**

We established a strategy that combined UC-MSC infusion and FMD and was effective in treating T2D, which provided potential approaches for developing novel clinical T2D therapies.

**Supplementary Information:**

The online version contains supplementary material available at 10.1186/s13287-021-02467-7.

## Introduction

It is estimated that 415 million people worldwide are currently living with diabetes [1], resulting in plenty of patients with complications in the liver, kidney, the cardiovascular system, and so on. Particularly, high-fat diet (HFD) results in the development of obesity which is a major risk factor for type 2 diabetes (T2D) [2, 3]. Since the liver is a major organ for glucose metabolism, it has become one of the important targets in maintaining the blood glucose homeostasis and in treating T2D. Noteworthy is that almost all patients with T2D display lipid accumulation in the liver causing hepatic steatosis, which represents a big hurdle to cure T2D [4, 5].

Fasting-mimicking diet (FMD), a kind of caloric restriction which represents a dietary mode low in calories, sugars, and proteins but high in unsaturated fats, can dramatically reduce triglycerides (TG) and total and low-density lipoprotein cholesterol, resulting in a loss of total body fat and a reduction of liver fat accumulation [6, 7]. Although some studies documented that FMD ameliorated T2D by inhibiting the inflammatory cytokines such as interleukin (IL)-1β, IL-4, IL-6, and tumor necrosis factor (TNF)-α [8, 9], its capacity of glycemic control may be unstable, and sometimes may even cause hypoglycemia, long-term treatment must be performed to achieve the expected therapeutic effects [6, 10].

Mesenchymal stem cells (MSCs) are a subset of multipotent stem cells with the capacity of immunomodulation/anti-inflammation through secreting cytokines, which makes them promising candidates for stem cell-based therapy [11, 12]. Previous studies have reported that systemic infusion of umbilical cord-derived MSCs (UC-MSCs) can not only reduce blood glucose and the incidence of diabetic complications in T2D patients [13], but also regulate immune reactions in diabetes by secreting cytokines, such as prostaglandin E2 (PGE2), nitric oxide (NO), transforming growth factor (TGF)-β, and hepatic growth factor (HGF), which inhibit proliferation and activation of T cells [14, 15]. Unfortunately, the effect on improving lipid metabolism disorder is not quite satisfactory [16].

Here, we aimed to investigate the efficacy comparison between FMD and UC-MSCs in the treatment of T2D in diet-induced obese (DIO) mouse model and discovered that FMD significantly reduced weight gain and improved the lipid metabolism while the effect on regulation of glucose metabolism was uncertain. Despite that therapeutic effect of UC-MSCs on the abnormal lipid metabolism in DIO mice was not good, it significantly ameliorated glucose disposal. These results motivated us to examine the treatment in DIO mice and leptin receptor-deficient (db/db) mice with the combination of UC-MSCs and FMD. It is revealed that UC-MSCs promoted liver function based on immunoregulation, which further enhanced the effects of FMD on controlling hyperglycemia and lipid metabolism disorders. The results showed great potential for new clinical T2D therapies.

## Methods

### Animal model

Four-week-old male C57/BL6 mice were purchased from Fourth Military Medical University (Xi’an, China) and 5-week-old male diabetic db/db (BKS.Cg-Dock7^m^ +/+ Lepr^db^/J) mice and non-diabetic m/m mice as normal group were purchased from Model Animal Research Center of Nanjing University (Nanjing, China). All mice are housed with a 12-h/12-h light/dark cycle at an ambient temperature of 22–25 °C. Animal studies were approved by Ethics Committee of Health Science Center, Xi’an Jiaotong University. All procedures were performed in accordance with the institutional guidelines for animal care and utilization. Five-week-old C57/BL6 mice were fed with high-fat diet (HFD, D12492, Research Diets, USA) to induce DIO mouse model for 16 weeks, and mice fed with regular chow diet (RCD) were used as control group.

### FMD treatment

The mouse FMD protocol is a 4-day regimen [17]. Each FMD cycle entails 4-day FMD and 7 days of refeeding (RF), which forms 11 days per cycle for 7 cycles. The FMD diet is 50% of the standard daily calorie intake on day 1 and 10% of normal daily calorie intake on day 2 to 4. Prior to supplying the FMD diet, animals were moved into fresh cages to avoid feeding on residual chow and coprophagy. All mice were supplied with wholesome food during the morning hours (8 a.m.–10 a.m.). FMD mice generally consumed the supplied food within the first few hours of the light cycle. Control-fed animals usually consumed the supplied food during the dark hours. All animals had access to water at all time.

### Cell culture, identification, and infusion

UC-MSCs were isolated from human umbilical cords freshly obtained from women who gave birth in Xi’an No. 4 Hospital. All of the subjects provided informed consent. Ethics approval was obtained from the Ethics Committee of Xi’an Fourth Hospital. UC-MSCs were isolated using the tissue block culture attachment method [18, 19]. In brief, umbilical cord vein and arteries with their surrounding Wharton jelly were separated from stroma by manual stripping. The mesenchymal tissue in Wharton jelly was minced into cubes of 2–3 mm^3^ pieces, transferred to petri dishes and cultured in a 37 °C incubator with 5% CO_2_ in minimum essential medium-α (α-MEM, Invitrogen, USA) with 10% fetal bovine serum (FBS, Gibco, USA). Then, medium was changed every 2 days after plating and 10 days later, the tissue blocks were removed. When the cells reached 70–80% confluence, they were harvested and cultured at a density of 1 × 10^4^ cells/cm^2^. The cells in passage 5 were used for experiments.

For the cellular identification, UC-MSCs at passage 5 were gathered to assess surface antigens by flow cytometry analysis on CytoFLEX flow cytometer (Beckman Coulter, Brea, California, USA). UC-MSCs were incubated with the following fluorescent antibodies, all from eBioscience (San Diego, CA, USA): fluorescein isothiocyanate (FITC)-labeled CD14 (11-0149-42), CD19 (11-0199-42), CD73 (11-0739-42), human leukocyte antigen DR (HLA-DR) or phycoerythrin (PE)-labeled, CD34 (12-0349-42), CD45 (12-0459-42), CD90 (12-0909-42), CD105 (12-1057-42), or IgG (12-4714-82).

The DIO or db/db mice were randomly divided into four groups (n = 6/group): DIO or db/db, UC-MSCs, FMD, and UC-MSCs combined with FMD (UC-MSCs + FMD). UC-MSCs (1 × 10^6^ cells/dose) were suspended in 0.2 ml phosphate buffer saline (PBS) and injected into mice via the tail vein. The control groups were treated with an infusion of 0.2 ml PBS. The mice were treated with cell therapy for 3 times on day 1 beginning of FMD, 30, and 60. At day 77, the end of the study period, all mice were fasted for 6 h, and blood glucose and body weight were measured, then sacrificed.

### Blood glucose analyses

The mice were starved for 6 h before the measurement of blood glucose levels and body weight. Tail venous blood glucose levels were monitored with a gluco-meter ACC-CHEKA performa (Roche, Indianapolis, IN, USA). For intraperitoneal glucose tolerance tests (IPGTT), after 6 h of fasting, all mice were intraperitoneally injected with 2 g/kg glucose and blood glucose was drawn to measure at 0, 15, 30, 60, 90, and 120 min after glucose injection. Area under the curve (AUC) above baseline was calculated as an index of glucose tolerance.

### Biochemical and cytokine assays

The levels of hemoglobin (Hb) and glycosylated hemoglobin (HbA_1c_) and in whole blood were measured by enzyme-linked immunosorbent assay (ELISA) kits (FanKew, China). After blood was placed at room temperature for 1 h, the serum was collected by centrifugation at 3000×*g* for 10 min. The levels of serum insulin, IL-1β, IL-6, IL-10, TNF-α, interferon (IFN)-γ, and TGF-β were determined also using ELISA kits (Neobioscience, China). Serum total cholesterol (TC), TG, free fatty acid concentrations (FFA), alanine aminotransferase (ALT), and aspartate transaminase (AST) were measured by chemical test kits (Nanjing Jian Cheng Bioengineer Institute, China). All experimental procedures were performed according to the instructions.

### Histological analysis

The fresh liver, skin, and white adipose tissue samples were fixed in 4% paraformaldehyde, dehydrated by serial alcohol, and embedded in paraffin. Paraffin-embedded sections were stained with hematoxylin-eosin (H&E) according to the standard protocol. For evaluating lipid accumulation in the liver, samples were frozen embedded in optimal cutting temperature compound (OCT, Leica, Wetzlar, Germany), sliced into 8 μm, and then stained with 0.5% Oil Red O solution for 30 min at room temperature. Quantification of lipid droplet area, thickness of skin fat layer and adipocyte size used the Image J software (National Institute of Mental Health, USA).

### Statistical analysis

All data were expressed as mean ± standard deviation (SD). The data were analyzed using unpaired two tailed Student *t*-tests for two group comparisons. One-way analysis of variance (ANOVA) follow by Tukey’s multiple comparison test was performed for multiple group comparisons. IPGTT were measured by two-way ANOVA follow by Tukey’s multiple comparison test. A value of *P* < 0.05 was considered significant. All statistical analyses were performed using GraphPad Prism 7.0 software (GraphPad Software, La Jolla, CA, USA).

## Results

### Identification of DIO mice model

**Fig. 1:**
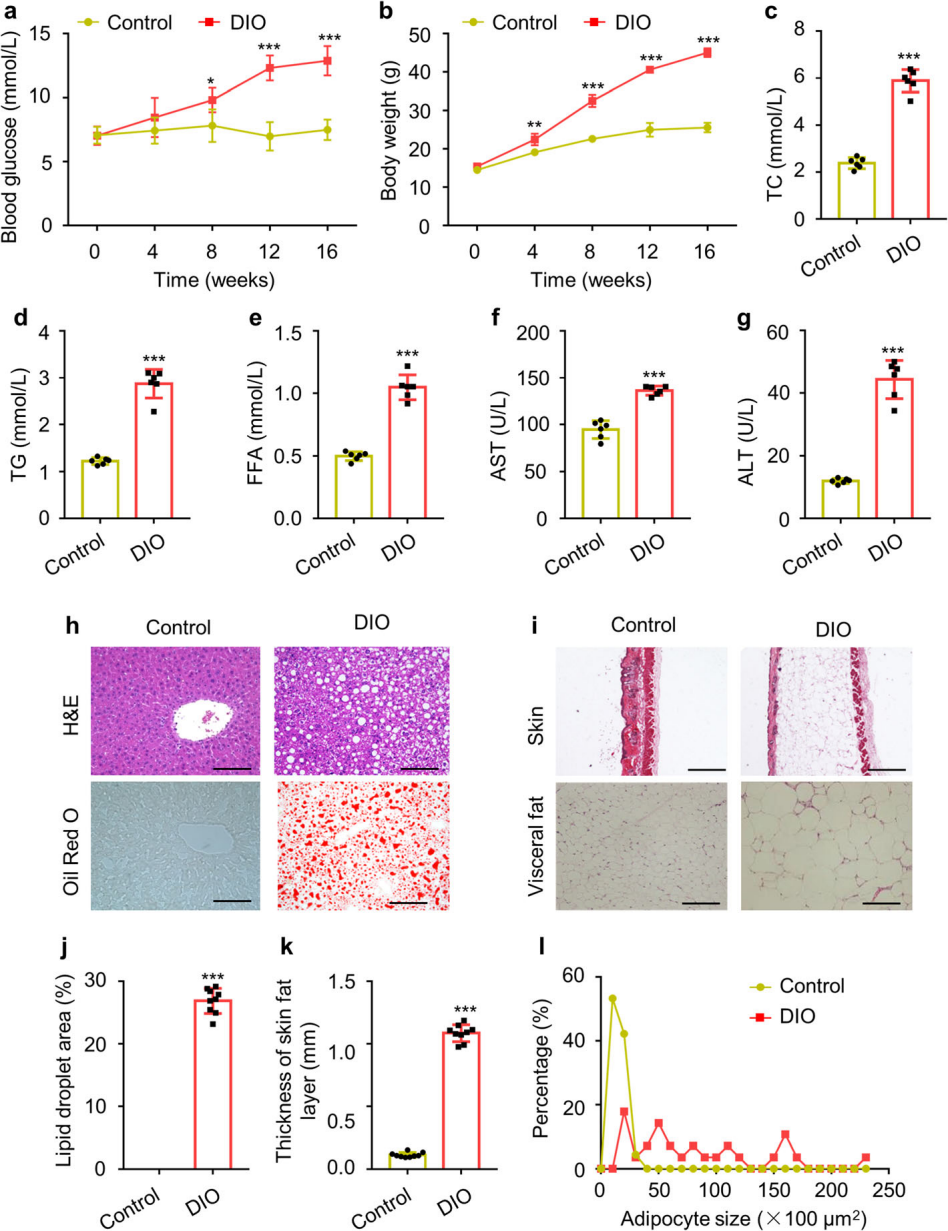
Metabolic studies in control and DIO mice. **a**, **b** Fasting blood glucose levels and body weight were determined every 4 weeks within 16 weeks. **c**–**g** The levels of serum TC, TG, FFA, AST, and ALT in control and DIO mice were detected by ELISA. **h** Liver steatosis were analyzed through staining with H&E (scale bar, 100 μm) and Oil Red O (scale bar, 200 μm). **i** H&E staining of skin (scale bar, 1 mm) and visceral fat (scale bar, 100 μm). **j** The quantification of lipid accumulation in **h**. **k** The quantification of thickness of subcutaneous fat layer in **i**. **l** The quantification of visceral adipocytes size in **i**. The data are expressed as mean values ± SD. n = 6 mice per group. *P < 0.05, **P < 0.01, ***P < 0.001

To test the therapeutic effects of UC-MSCs and FMD cycles on T2D, DIO mouse model was induced by 16 weeks of HFD feeding. With HFD feeding, blood glucose levels in DIO mice increased gradually and were significantly higher than the control group from 8 weeks of HFD feeding (Fig. 1a). Then, IPGTT showed noticeable deterioration of glucose disposal in DIO mice (Figure S1a, b). The levels of HbA_1c_ and serum insulin of DIO mice increased markedly (Figure S1c-e). Besides, after 16 weeks of HFD feeding, DIO mice showed significantly increased body weight (Fig. 1b) and the body weight gain was 18 g more than of the control group (Figure S1f). Moreover, the concentrations of serum AST, ALT, TC, TG, and FFA were substantially upregulated in DIO mice, indicating dysregulated lipid metabolism and impaired liver function (Fig. 1c–g). In addition, H&E and Oil Red O staining of liver indicated severe hepatic steatosis in the DIO mice, shown as the accumulation of lipid droplets in hepatocytes (Fig. 1h, j). Thickened skin fat layer and larger visceral adipocyte size were presented by H&E staining of skin and visceral fat (Fig. 1i, k, l). These results showed successful establishment of the DIO type 2 diabetic mouse model.

### UC-MSC infusion improved glucose homeostasis in DIO mice better than FMD cycles

For identification of UC-MSCs, they were be harvested, and the cell phenotypes were detected using flow cytometry. UC-MSCs expressed CD73, CD90, and CD105, and seldom expressed CD14, CD19, CD34, CD45, or HLA-DR, which were consistent with the phenotypical characteristics of MSCs (Fig S2a).

**Fig. 2:**
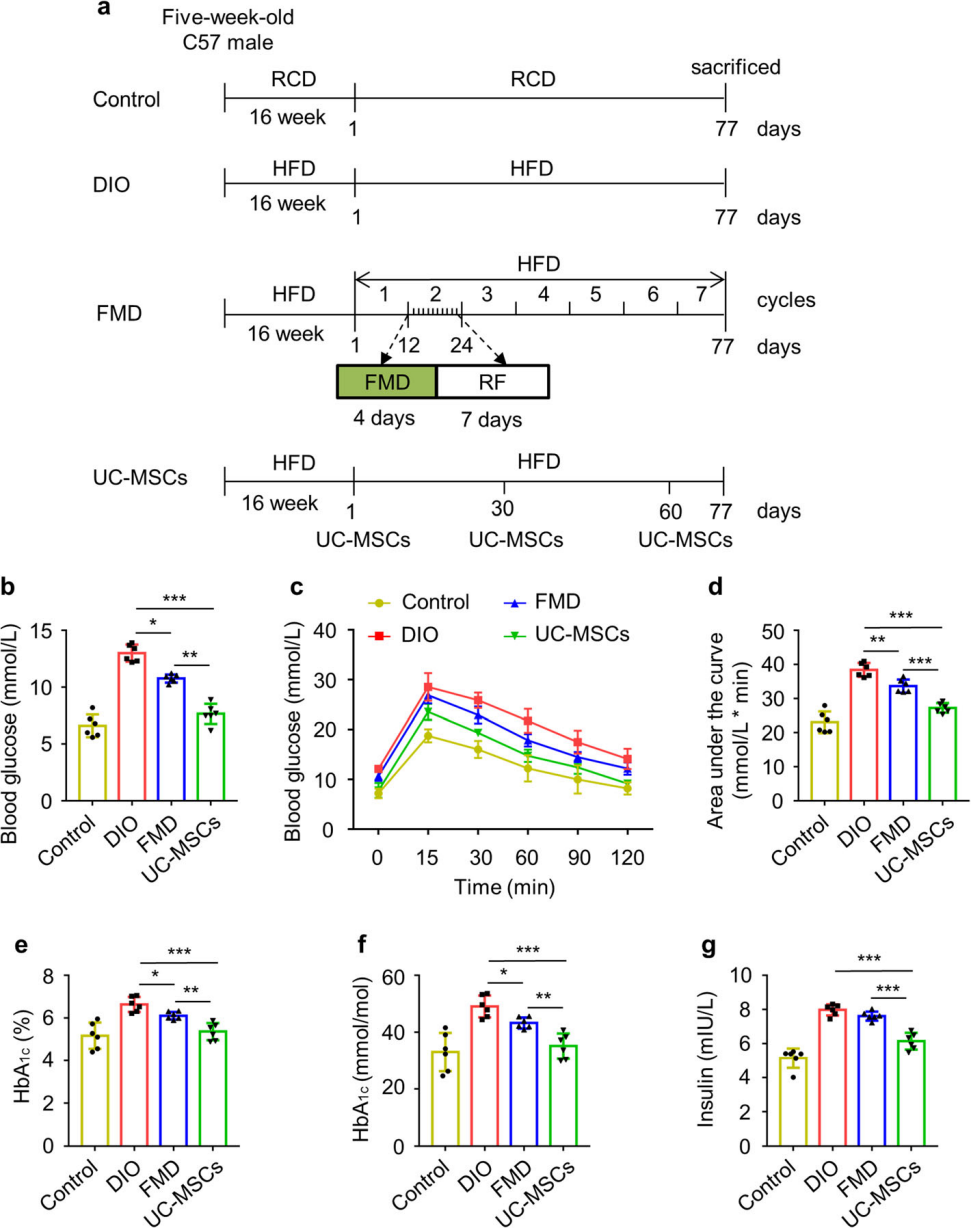
UC-MSC infusion improved glucose homeostasis in DIO mice better than FMD cycles. **a** Experimental scheme to determine effects of the periodic FMD and UC-MSCs on DIO mice. Each FMD cycle entails 4 days FMD and 7 days of refeeding (RF), which forms 11 days per cycle for 7 cycles. During refeeding, mice received a HFD identical to that given prior to the FMD. The control and UC-MSC groups have access to ad libitum feeding. **b** Fasting blood glucose levels were monitored after fasting 6 h at sacrificed. **c**, **d** Glucose tolerance was assessed by IPGTT. AUC above baseline was calculated as an index of glucose tolerance. **e**–**g** ELISA analyzed HbA_1c_, Hb, and serum insulin. The data are expressed as mean values ± SD. n = 6 mice per group. *P < 0.05, **P < 0.01, ***P < 0.001

To investigate the therapeutic effects of UC-MSC infusion and FMD cycles on DIO mice, DIO mice were divided into three groups: DIO, FMD, and UC-MSCs (1 × 10^6^ cells/dose in 0.2 mL PBS) (Fig. 2a). With treatment, fasting blood glucose in FMD (10.8 ± 0.36 mmol/L) and UC-MSCs (7.7 ± 0.89 mmol/L) group were decreased and lower than DIO group (13.0 ± 0.75 mmol/L), but fasting blood glucose in the FMD group was still a little high (Fig. 2b). The results of IPGTT showed UC-MSC infusion and FMD cycles could ameliorate glucose disposal with reduced AUC in DIO mice, but the UC-MSC group was better than the FMD group (Fig. 2c, d). Besides, HbA_1c_ and serum insulin concentration were not markedly changed after receiving 7 FMD cycles but dramatically declined after UC-MSC infusion (Fig. 2e–g).

### FMD cycles regulated lipid metabolism in DIO mice better than UC-MSC infusion

**Fig. 3:**
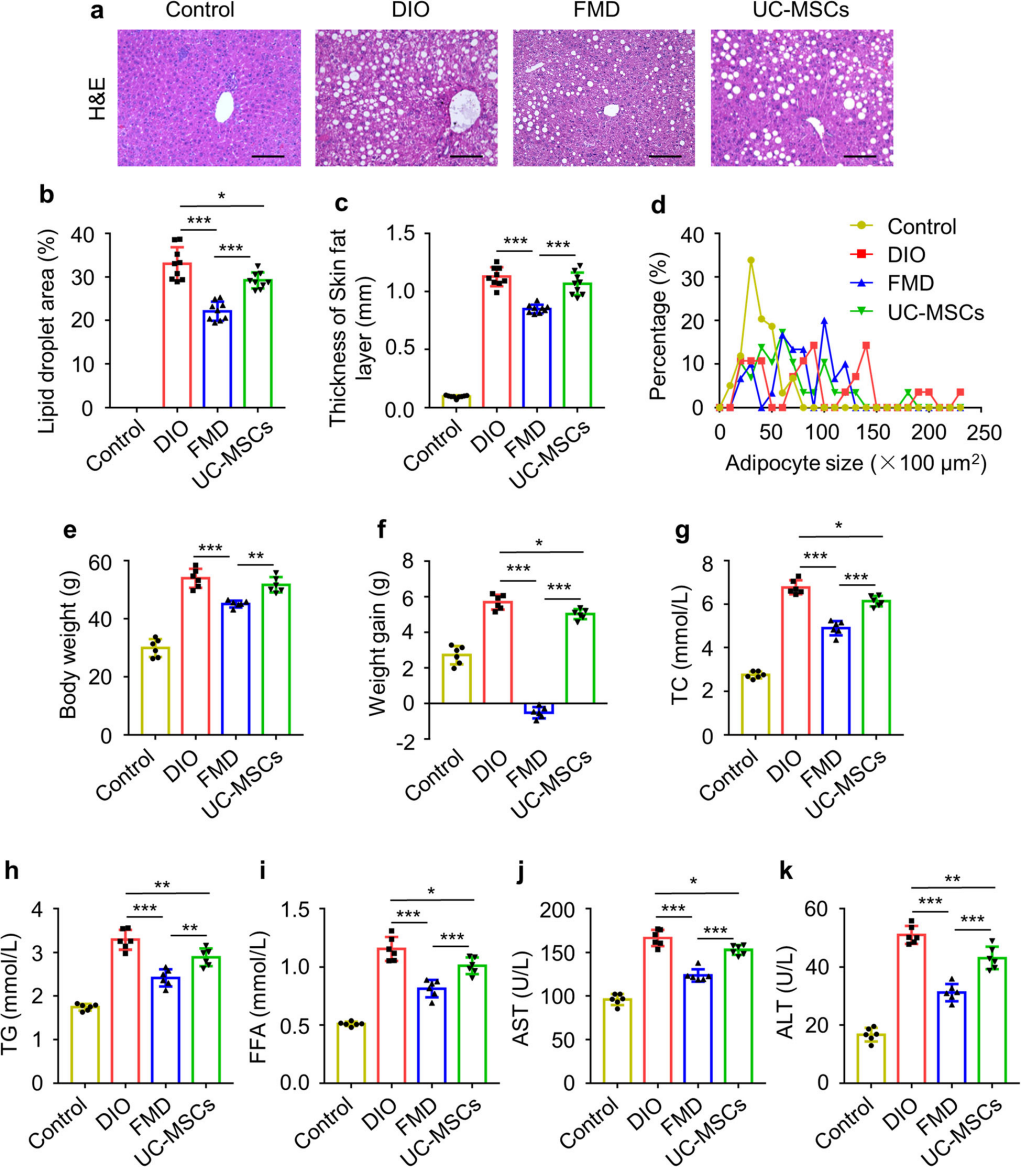
FMD cycles regulated lipid metabolism in DIO mice better than UC-MSC infusion. **a** Liver steatosis were analyzed through staining with H&E (scale bar, 100 μm). **b** The quantification of lipid accumulation in **a**. **c** Quantification of thickness of subcutaneous fat layer. **d** Quantification of visceral adipocytes size. **e**, **f** Body weights were determined after fasting 6 h at sacrificed. **g**–**k** The levels of serum TC, TG, FFA, AST, and ALT were detected by ELISA. The data are expressed as mean values ± SD. n = 6 mice per group. *P < 0.05, **P < 0.01, ***P < 0.001

H&E and Oil red O staining showed the FMD cycles markedly reduced the liver steatosis, but only slight alleviation of liver steatosis by UC-MSC infusion (Fig. 3a, b, Figure S3a). Moreover, the skin fatty layer thickness and the visceral adipocyte size were reduced by FMD cycles and no substantially change was observed after UC-MSC infusion, as examined by H&E (Fig. 3c, d, Figure S3b, c). Furthermore, after receiving 7 FMD cycles, the FMD group showed a significant declination in body weight (45.06 ± 1.18 g) compared to the DIO mice (53.96 ± 3.27 g) (Fig. 3f), together with weight loss (− 0.52 ± 0.41 g) (Fig. 3e). Body weight in the UC-MSC group (51.72 ± 2.64) had a lower weight gain (5.01 ± 0.28 g) than DIO group (5.70 ± 0.41 g) while no significant difference was detected between two group in body weight (Fig. 3e, f). In addition, the concentrations of serum TC, TG, FFA, AST, and ALT in DIO mice were suppressed after FMD cycles and UC-MSC infusion and FMD cycles were much better than UC-MSC infusion (Fig. 3g–k). These results demonstrated effective effect of FMD on the lipid metabolism in T2D but uncertain effect on hyperglycemia, and effective glycemic control by UC-MSCs with scarce effect on the lipid metabolism.

### UC-MSC infusion combined with FMD improved glucose homeostasis as UC-MSC infusion

**Fig. 4:**
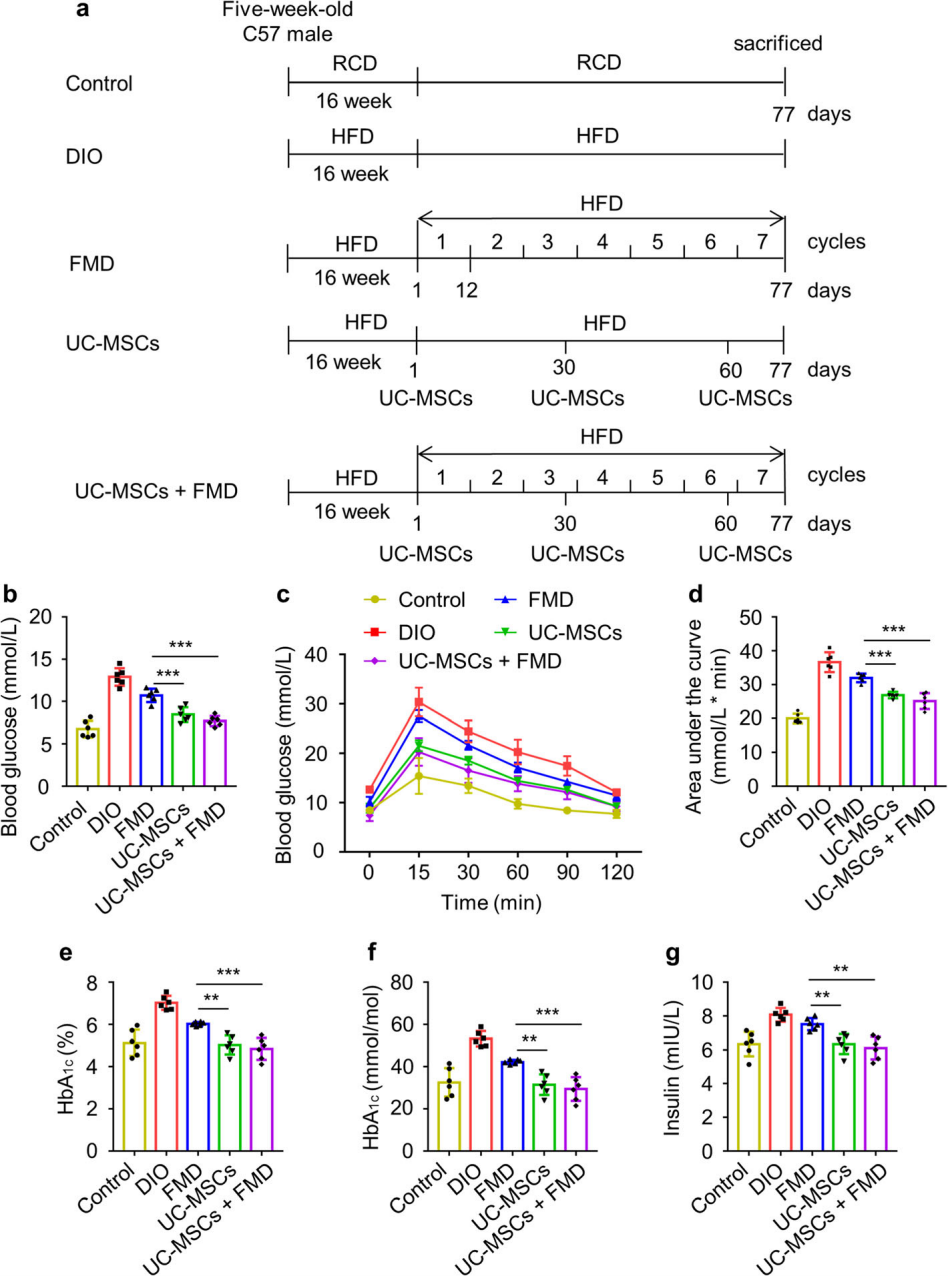
UC-MSCs combined with FMD improved glucose homeostasis as UC-MSC infusion. **a** Experimental scheme to determine effects of the periodic FMD and UC-MSCs on DIO mice. Each FMD cycle entails 4 days FMD and 7 days of refeeding (RF), which forms 11 days per cycle for 7 cycles. During refeeding, mice received a HFD identical to that given prior to the FMD. The control and UC-MSC groups have access to ad libitum feeding. **b** Fasting blood glucose levels were monitored after fasting 6 h at sacrificed. **c**, **d** Glucose tolerance was assessed by IPGTT. AUC above baseline was calculated as an index of glucose tolerance. **e**–**g** ELISA analyzed HbA_1c_, Hb, and serum insulin. The data are expressed as mean values ± SD. n = 6 mice per group. *P < 0.05, **P < 0.01, ***P < 0.001

To evaluate whether UC-MSCs combined with FMD can further improve glucose homeostasis in DIO mice, we compared the fasting blood glucose levels of DIO mice treated with FMD, UC-MSCs (1 × 10^6^ cells/dose in 0.2 mL PBS) and UC-MSCs combined with FMD (Fig. 4a). We found that fasting blood glucose in UC-MSCs combined with the FMD group (7.7 ± 0.60 mmol/L) was decreased and lower than the FMD group (10.7 ± 0.79 mmol/L), as no significant difference with the UC-MSC group (8.47 ± 0.85 mmol/L) (Fig. 4b). The results of IPGTT further showed UC-MSCs combined with FMD ameliorate glucose tolerance of DIO mice more than FMD cycles and no significant difference with UC-MSC infusion (Fig. 4c, d). Moreover, HbA_1c_ and serum insulin concentration remarkably declined by UC-MSCs combined with FMD, better than FMD cycles, like UC-MSC infusion (Fig. 4e–g). These results provided UC-MSCs combined with FMD same as UC-MSC infusion in maintaining glucose homeostasis.

### UC-MSCs combined with FMD regulated lipid metabolism better than FMD cycles

**Fig. 5:**
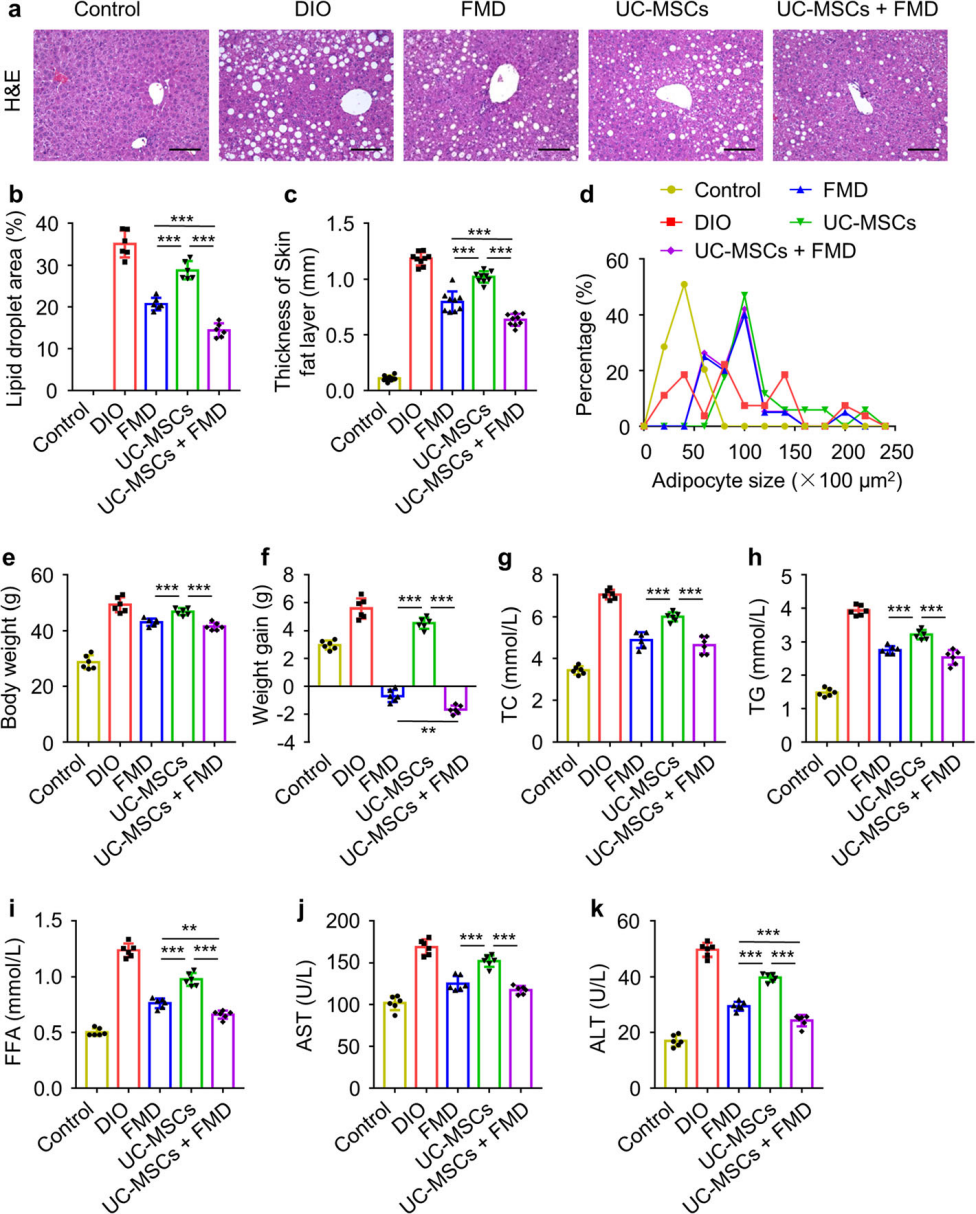
UC-MSCs combined with FMD regulated lipid metabolism better than FMD cycles. **a** Liver steatosis were analyzed through staining with H&E (scale bar, 100 μm). **b** The quantification of lipid accumulation in **a**. **c** Quantification of thickness of subcutaneous fat layer. **d** Quantification of visceral adipocytes size quantification. **e**, **f** Body weights were determined after fasting 6h at sacrificed. **g**–**k** The levels of serum TC, TG, FFA, AST, and ALT were detected by ELISA. The data are expressed as mean values ± SD. n = 6 mice per group. *P < 0.05, **P < 0.01, ***P < 0.001

To investigate whether UC-MSCs combined with FMD can further regulate lipid metabolisms of DIO mice, we analyzed the histological change of liver, skin, and visceral fat. As expected, UC-MSCs combined with FMD markedly reduced the liver steatosis in DIO mice as same as FMD cycles and better than UC-MSC infusion, as examined by H&E and Oil red O staining (Fig. 5a, b, Figure S4a). Consistently, the skin fatty layer thickness and the visceral adipocyte size were reduced by UC-MSCs combined with FMD more than FMD cycles and UC-MSC infusion (Fig. 5c, d, Figure S4b, c). Besides, UC-MSCs combined with FMD and FMD cycles both resulted in a reduction in HFD-fed body weight, and the weight loss in UC-MSCs combined with the FMD group (− 1.68 ± 0.30 g) was more than the FMD group (− 0.69 ± 0.44 g) (Fig. 5e, f). Meanwhile, the levels of serum TC, TG, FFA, AST, and ALT in UC-MSCs combined with the FMD group were considerably decreased and lower than the UC-MSC group (Fig. 5g–k). What is more, the levels of serum TC, TG, and AST in UC-MSCs combined with the FMD group had no significant difference with the FMD group, but the levels of serum FFA and ALT in UC-MSCs combined with the FMD group was substantially lower than the FMD group (Fig. 5g–k). These results suggested UC-MSCs combined with FMD better than FMD cycles in restoring lipid metabolisms.

### UC-MSCs combined with FMD suppressed inflammation in DIO mice

**Fig. 6:**
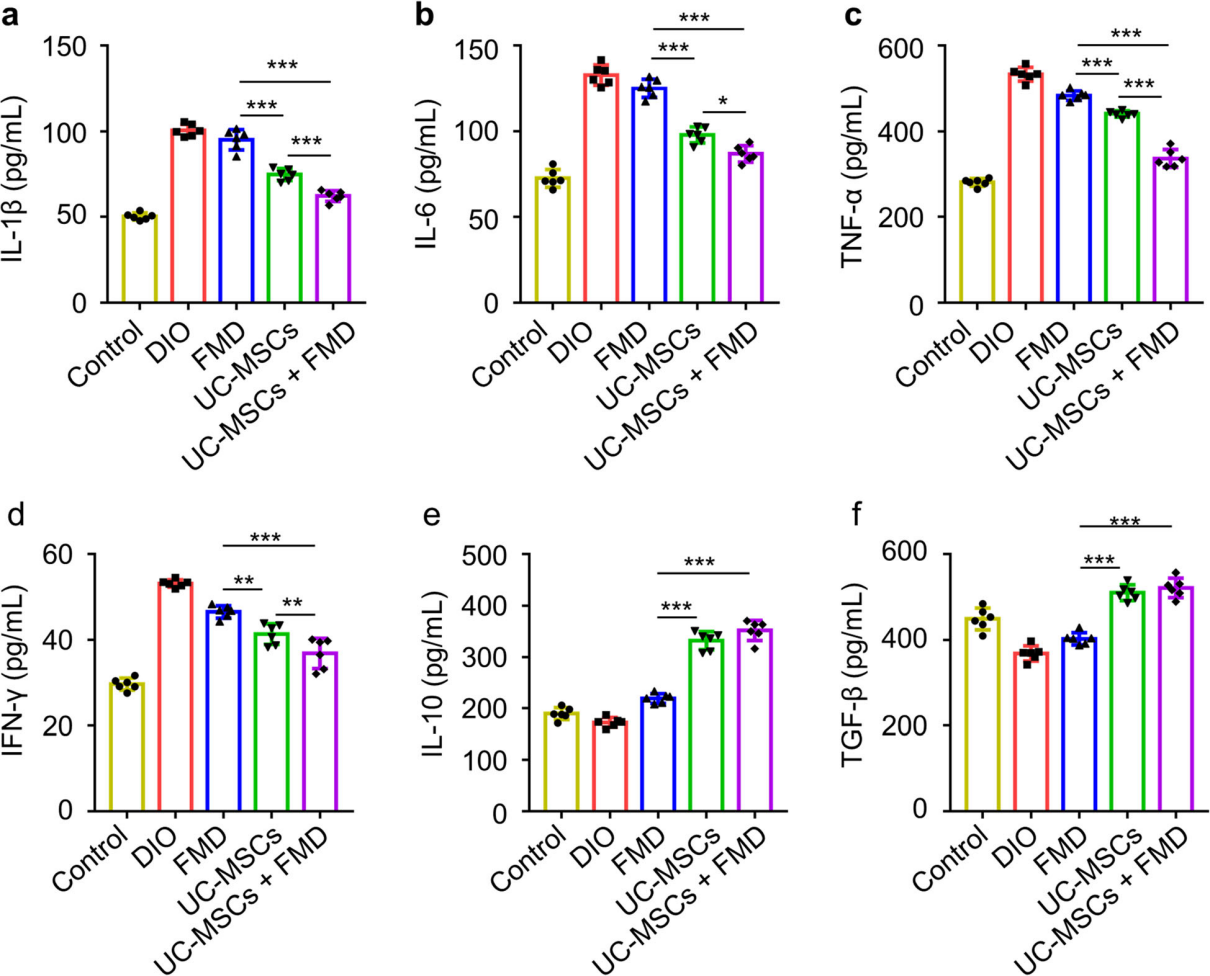
UC-MSCs combined with FMD suppressed inflammation. **a**–**f** ELISA analysis of serum IL-1β, IL-6, TNF-α, IFN-γ, IL-10, and TGF-β in control, DIO, FMD, UC-MSCs, and UC-MSCs + FMD groups. The data are expressed as mean values ± SD. n = 6 mice per group. *P < 0.05, **P < 0.01, ***P < 0.001

To explore the mechanisms underlying synergistic effect of FMD and UC-MSCs, we analyzed the serum inflammation cytokine levels in different groups. The concentrations of proinflammatory cytokines IL-1β (Fig. 6a) and IL-6 (Fig. 6b) were dramatically reduced by UC-MSC infusion and UC-MSCs combined with FMD, especially UC-MSCs combined with FMD which lower than the UC-MSC group, while they did not change significantly after FMD cycles. However, the concentrations of proinflammatory cytokines TNF-α and IFN-γ were decreased by FMD cycles, UC-MSC infusion, and UC-MSCs combined with FMD (Fig. 6c, d). Compared with the FMD group, UC-MSCs and UC-MSCs combined with the FMD groups were lower than the FMD group, together with UC-MSCs combined with the FMD group were lower than the UC-MSC group (Fig. 6c, d). Moreover, FMD cycles, UC-MSC infusion, and UC-MSCs combined with FMD all increased the concentration of the anti-inflammatory cytokine IL-10, but UC-MSCs combined with FMD were higher than the FMD group and no significant difference with the UC-MSC group (Fig. 6e). Besides, the concentrations of serum TGF-β were increased by UC-MSC infusion and UC-MSCs combined with FMD, with no change in the FMD group, and no significant difference was detected between UC-MSC infusion and UC-MSCs combined with the FMD group (Fig. 6f). These results displayed immunoregulatory function by UC-MSCs contributed to strengthen actions of FMD in T2D therapy.

### UC-MSCs combined with FMD regulated lipid metabolism in db/db mice

To confirm the therapeutic effect of UC-MSCs combined with FMD on T2D, we used db/db mice to verify above results. The db/db mice were divided into four groups: db/db, FMD, UC-MSCs (1 × 10^6^ cells/dose in 0.2 mL PBS), and UC-MSCs combined with FMD. The fasting blood glucose in UC-MSCs combined with the FMD group (7.4 ± 1.41 mmol/L) was lower than that in the FMD group (10.6 ± 0.81 mmol/L), and no significant difference with the UC-MSC group (6.7 ± 1.22 mmol/L) (Figure S3a). Besides, FMD cycles, UC-MSC infusion, and UC-MSCs combined with FMD all could control weight gain in db/db mice, FMD cycles (3.08 ± 0.96 g), and UC-MSCs combined with FMD (2.41 ± 1.29 g) were better than UC-MSC infusion (4.84 ± 0.81 g) (Figure S3b. c).

**Fig. 7:**
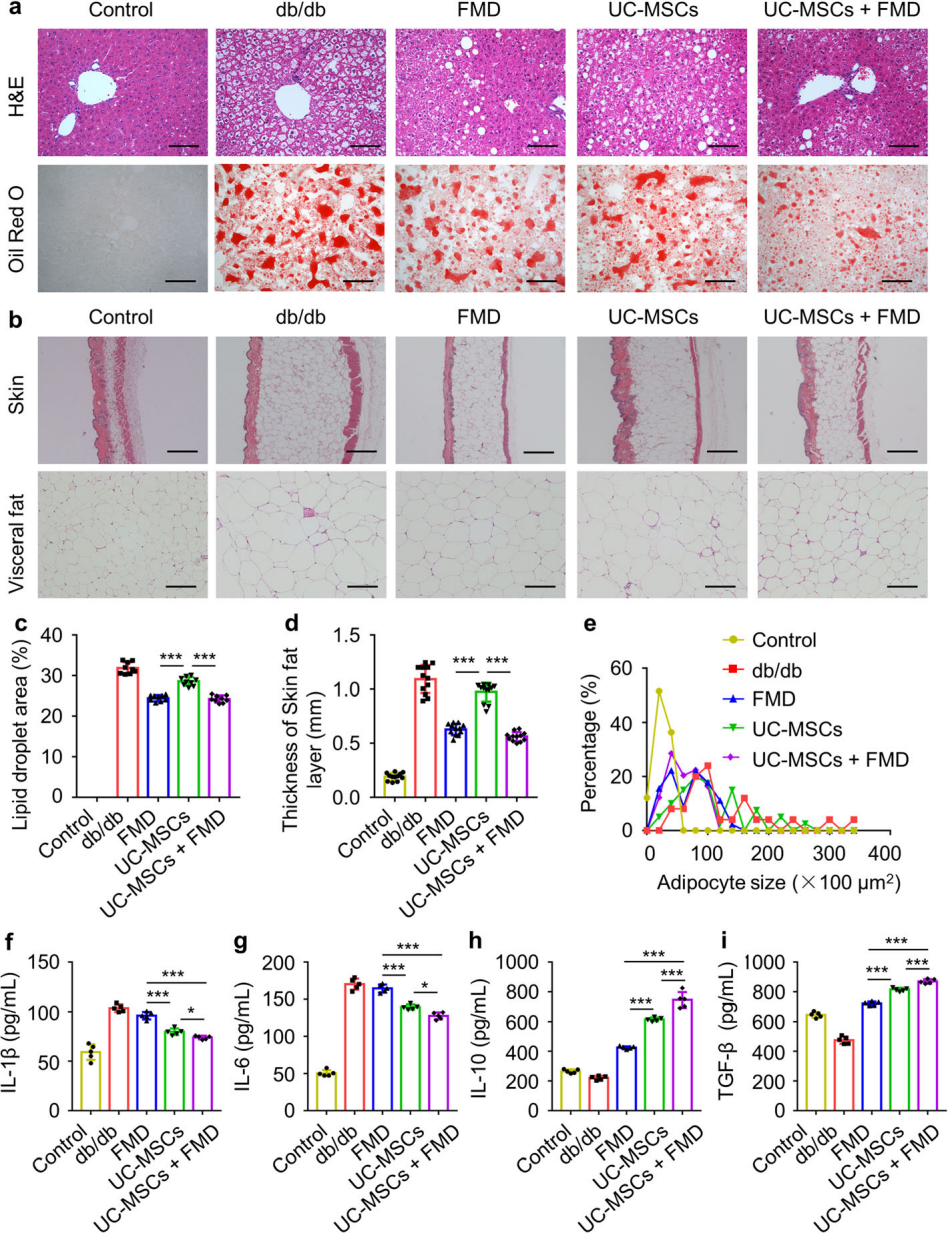
UC-MSCs combined with FMD regulated lipid metabolism in db/db mice. **a** Liver steatosis were analyzed through staining with H&E (scale bar, 100 μm) and Oil Red O (scale bar, 200 μm). **b** H&E staining of skin (scale bar, 1 mm) and visceral fat (scale bar, 100 μm). **c** The quantification of lipid accumulation in **a**. **d** Thickness of subcutaneous fat layer quantification in **b**. **e** Visceral adipocytes size quantification in **b**. **f**–**i** The levels of serum IL-1β, IL-6, IL-10, and TGF-β were detected by ELISA. The data are expressed as mean values ± SD. n = 6 mice per group. *P < 0.05, **P < 0.01, ***P < 0.001

Same as DIO mice model, the histopathological results supported by H&E and Oil red O staining in db/db mice showed liver steatosis, thickened skin fat layer, and larger visceral adipocyte size in UC-MSCs combined with the FMD group, in comparison with FMD cycles and UC-MSC infusion group (Fig. 7a–e). For the inhibition of proinflammatory cytokines IL-1β and IL-6 and the promotion of anti-inflammatory cytokine IL-10 and TGF-β, the results obtained in db/db mice model were consistent with those of DIO mice model (Fig. 7f–i).

## Discussion

FMD has recently been tested in the research of prediabetic and diabetic treatment and has shown immense therapeutic potential particularly for controlling dyslipidemia, but there are still some deficiencies regarding it effects on the glucose metabolism [6, 10]. Here, we investigated the effects of FMD and UC-MSC infusion on DIO type 2 diabetic mice and discovered that FMD indeed reduced weight gain and restored the lipid metabolism, but its capacity of controlling blood glucose was questionable. UC-MSC infusion can markedly decrease hyperinsulinemia and realized glycemic control; nevertheless, it had a little effect on dyslipidemia. Further experiments using combined UC-MSC infusion and FMD demonstrated effective regulation both of glucose and lipid metabolisms of DIO and db/db mice, which was based on immunoregulation of UC-MSCs. These results shed light on a promising approach to T2D with translational potential.

Diabetes mellitus is a chronic metabolic disease which requires continuous medical care and multi-factorial risk reduction strategies. T2D, which accounts for 90% of diabetic prevalence, encompasses individuals who have insulin resistance and relative insulin deficiency [20]. It has been reported that calorie restriction or changes in dietary composition can induce a specific lipidome and metabolome reprogramming event in liver, which may have positive effect on diabetic treatment [21, 22]. FMD, as a special intermittent fasting diet, can dramatically reduce TG and total and low-density lipoprotein cholesterol, resulting in a loss of total body fat reported by previous studies [6, 7]. One study found that lipogenesis pathway and ketogenesis pathway enzymes in the liver of diabetic mice were reduced by dietary interventions. In addition, FMD reversed the enhanced autophagy, mitochondrial biogenesis, collagen deposition, and endoplasmic reticulum stress in diabetic mice [23]. However, there are also reports claiming that mice receiving the alternate-day fasting regimen are more tolerant of glucose on the feeding day than on the fasting day, indicating that fasting may also impair the normal glucose metabolism. Meanwhile, the fasting blood glucose level fluctuated significantly during FMD [10, 24].

UC-MSCs are highly multipotent stem cells expressing markers such as Oct-4, Sox-2, and Nanog [25]. There are increasing evidence indicating the therapeutic effects of UC-MSC transplantation in a spectrum of diseases, including spinal cord injury, colitis, and myocardial infarction, due to its capacity of secreting various cytokines and growth factors [26–28]. Furthermore, recent studies have unveiled that UC-MSC infusion can potently promote beta-cell function, which might be correlated with tissue repair or cytoprotective properties of MSCs [29]. They can also reverse insulin resistance and improve islet function by suppressing NLRP3 inflammasome-mediated inflammation and eliciting macrophages into an anti-inflammatory phenotype [30], underlying their effects to lower blood glucose and HbA_1c_ without immediate or delayed toxicity. However, there have been studies reported that the effect of UC-MSCs on improving lipid metabolism disorder is not quite satisfactory [16].

DIO mouse is an acquired obesity model induced by high-fat diet, which often appear with obesity and diabetes-related symptoms [31]. In this study, we discovered that FMD cycles were effective in treating lipid metabolic disorders in DIO type 2 diabetic mice. But the capacity of FMD to reverse alterations in glucose homeostasis was indeed inferior than UC-MSCs, adding to the current knowledge of FMD efficacy on T2D. We also confirmed the glycemic control efficacy of UC-MSCs, while UC-MSC infusion did have less influences on ameliorating weight gain and dyslipidemia than FMD in T2D, which provided intriguing evidence for rethinking the translational strategy. These phenomena enlightened us to consider the treatment of diabetes with the combination of UC-MSCs and FMD. As proved here, UC-MSCs promoted FMD effects on ameliorating hyperglycemia and restoring the lipid metabolism in DIO type 2 diabetic mice, while FMD had little promotion effect on UC-MSCs.

It is not a straightforward process to generalize results from studies conducted in mice to the human clinical populations, taking into account differences in body mass, feeding patterns, behaviors, and other interventions between mice and humans [32]. There was a clinical trial reported that intermittent fasting reduced fasting insulin and insulin resistance [33], while another study found that although intermittent fasting groups lost weight, fasting glucose, fasting insulin, and insulin resistance were not improved [34]. Therefore, it is necessary to develop personalized intermittent fasting methods according to the unique metabolomic characteristics of each individual.

Immune dysfunction has been increasingly recognized as an important pathogenesis of T2D, in which long-term activation of both innate and adaptive immune responses leads to chronic systemic inflammation contributing to insulin resistance and relative insulin deficiency [35]. In this regard, elevated levels of circulating inflammatory markers have been considered a hallmark of T2D and an aggravation mechanism for its progression [36]. Several studies have shown the main target cells of inflammation to develop insulin resistance in T2D are adipocytes [37]. There is also evidence showing that proinflammatory cytokines including TNF-α, IL-1β, and IFN-γ disrupt the regulation of intracellular calcium in beta cells, thereby inhibiting the release of insulin. In addition, TNF-α increases the expression of islet amyloid polypeptide in beta cells, resulting in accelerated death [38]. Given the important role of inflammation during the progression of diabetes, there are oral and injectable therapies being developed. For one instance, many studies have demonstrated that intensive insulin therapy can significantly downregulate serum IL-2, TNF-α, INF-γ, and IL-4 concentrations with upregulation of IL-10 in diabetic patients, contributing to the anti-inflammatory status in treating these patients [39]. Metformin is one of the recommended first-line glucose-lowering medications for treating T2D [40]. Notably, metformin can reduce inflammatory cytokines, such as TNF-α, IL-6, and IL-1 and induce the production of anti-inflammatory cytokines, such as IL-4 and IL-10 [41]. MSCs also exert the potent ability to ameliorate systemic inflammation and restore the homeostasis of the immune microenvironment, in which they reduce serum proinflammatory cytokines, including IL-6, IL-1α, IL-1β, and IFN-γ, and promote epidermal growth factor (EGF) and IL-10 [42]. Here, we further confirmed immunoregulatory effect of UC-MSCs on T2D, which may serve as a pivotal mechanism underlying their therapeutic effects. Particularly, the effect of FMD on inflammatory responses was significantly increased in combination with UC-MSCs; these phenomena in reducing circulating proinflammatory mediators and elevating anti-inflammatory cytokines may represent the normalization of immune function to a balanced status, thus decreasing systemic inflammatory response and restoring normal insulin resistance and relative insulin deficiency.

db/db mice are a type of spontaneous obese diabetic mice whose leptin receptor mutation leads to leptin signaling pathway dysfunction; they also appear with obesity and diabetes-related symptoms such as insulin resistance, significant increases in blood glucose levels, and hepatic steatosis [43]. We finally demonstrated the role of UC-MSCs combined with FMD in db/db mouse model. Similar to DIO mice, it showed excellent capabilities in ameliorating hyperglycemia and regulating lipid metabolism. Since patients with T2D have a growing prevalence of overweight and dyslipidemia [44], based on the regulation of lipid metabolism by FMD, our strategy realized the purpose of further promoting the regulation of glucose metabolism by UC-MSCs, which ultimately provides a new idea for the treatment of T2D.

## Conclusions

We established a strategy that combined UC-MSC infusion and FMD were effective in treating T2D, which synergistically attenuated hyperglycemia and improved the lipid metabolism through immunoregulation. This work is of great significance for the development of novel clinical T2D approaches.

## Supplementary Information

**Additional file 1: Figure S1.** Glucose homeostasis in control and DIO mice. (a, b): Glucose tolerance was assessed by IPGTT. AUC above baseline was calculated as an index of glucose tolerance. (c-e): ELISA analyzed the levels of HbA_1c_, Hb and serum insulin. (f): weight gain after 16 weeks of HFD. The data are expressed as mean values ± SD. n=6 mice per group. *P < 0.05, **P < 0.01, ***P< 0.001. **Figure S2.** The identification of UC-MSCs. (a): Flow Cytometry results determining the UC-MSCs phenotype. UC-MSCs were stained with FITC-labeled CD14, CD19, CD73, HLA-DR and PE-labeled, CD34, CD45, CD90, CD105. **Figure S3.** Histomorphological changes of liver, skin and visceral fat. (a): Liver steatosis were analyzed through staining with Oil Red O (Scale bar, 200 μm). (b): H&E staining of skin (Scale bar, 1 mm). (c): H&E staining of visceral fat (Scale bar, 100 μm). **Figure S4.** Histomorphological changes of liver, skin and visceral fat. (a): Liver steatosis were analyzed through staining with Oil Red O (Scale bar, 200 μm). (b): H&E staining of skin (Scale bar, 1 mm). (c): H&E staining of visceral fat (Scale bar, 100 μm). **Figure S5.** The effect of UC-MSCs combined with FMD on blood glucose and body weight in db/db mice. (a-c): Blood glucose and body weight were determined after fasting 6h at sacrificed. The data are expressed as mean values ± SD. n=6 mice per group. *P < 0.05, **P < 0.01, ***P< 0.001.

## Data Availability

The datasets used and analyzed during the current study are available from the corresponding author on reasonable request.
