# Supplementary material for: Glycemic control by umbilical cord-derived mesenchymal stem cells promotes effects of fasting-mimicking diet on type 2 diabetic mice
Source: Stem Cell Res Ther. 2021 Jul 13;12:395. doi: 10.1186/s13287-021-02467-7 (PMC8278637; doi:10.1186/s13287-021-02467-7)
Supplement: Supplementary file 1 — Additional file 1: Figure S1. Glucose homeostasis in control and DIO mice. (a, b): Glucose tolerance was assessed by IPGTT. AUC above baseline was calculated as an index of glucose tolerance. (c-e): ELISA analyzed the levels of HbA1c, Hb and serum insulin. (f): weight gain after 16 weeks of HFD. The data are expressed as mean values ± SD. n=6 mice per group. *P < 0.05, **P < 0.01, ***P< 0.001. Figure S2. The identification of UC-MSCs. (a): Flow Cytometry results determining the UC-MSCs phenotype. UC-MSCs were stained with FITC-labeled CD14, CD19, CD73, HLA-DR and PE-labeled, CD34, CD45, CD90, CD105. Figure S3. Histomorphological changes of liver, skin and visceral fat. (a): Liver steatosis were analyzed through staining with Oil Red O (Scale bar, 200 μm). (b): H&E staining of skin (Scale bar, 1 mm). (c): H&E staining of visceral fat (Scale bar, 100 μm). Figure S4. Histomorphological changes of liver, skin and visceral fat. (a): Liver steatosis were analyzed through staining with Oil Red O (Scale bar, 200 μm). (b): H&E staining of skin (Scale bar, 1 mm). (c): H&E staining of visceral fat (Scale bar, 100 μm). Figure S5. The effect of UC-MSCs combined with FMD on blood glucose and body weight in db/db mice. (a-c): Blood glucose and body weight were determined after fasting 6h at sacrificed. The data are expressed as mean values ± SD. n=6 mice per group. *P < 0.05, **P < 0.01, ***P< 0.001. [file 13287_2021_2467_MOESM1_ESM.docx]

**Supplementary Data**

**Glycemic Control by Umbilical Cord-Derived Mesenchymal Stem Cells Promotes Effects of Fasting-Mimicking Diet on Type 2 Diabetic Mice**

Na Zhao^1,2†^, Ying-Feng Gao^1,2†^, Lei Bao^3†^, Jing Lei^3^, Huan-Xiao An^1^, Feng-Xing Pu^1^, Rui-Ping Cheng^1^, Ji Chen^4^, Hua Ni^1^, Bing-Dong Sui^4*^, Fan-Pu Ji^2,5,6*^, Cheng-Hu Hu^1,3*^

^1^ Institute for Stem Cell & Regenerative Medicine, The Second Affiliated Hospital of Xi'an Jiaotong University, Xi’an, Shaanxi, People’s Republic of China;

^2^ National & Local Joint Engineering Research Center of Biodiagnosis and Biotherapy, The Second Affiliated Hospital of Xi'an Jiaotong University, Xi’an, Shaanxi, People’s Republic of China;

^3^ Department of Obstetrics and Gynecology, Xi'an No. 4 Hospital, Affiliated Guangren Hospital, School of Medicine, Xi'an Jiaotong University, Xi'an, Shaanxi, People’s Republic of China;

^4^ State Key Laboratory of Military Stomatology & National Clinical Research Center for Oral Diseases & Shaanxi International Joint Research Center for Oral Diseases, Center for Tissue Engineering, School of Stomatology, The Fourth Military Medical University, Xi’an, Shaanxi, People’s Republic of China;

^5^ Department of Infectious Diseases, The Second Affiliated Hospital of Xi'an Jiaotong University, Xi’an, Shaanxi, People’s Republic of China;

^6^ Key Laboratory of Environment and Genes Related to Diseases, Xi'an Jiaotong University, Ministry of Education of China, Xi'an, Shaanxi, People’s Republic of China.

* Correspondence: **Dr. Cheng-Hu Hu** (E-mail: [chenghu@xiterm.com](mailto:chenghu@xiterm.com)) and **Dr. Fan-Pu Ji** (E-mail: [jifanpu1979@163.com](mailto:jifanpu1979@163.com)) 157 Xi Wu Road, Xi’an 710004, Shaanxi, China. **Dr. Bing-Dong Sui** (Email: [bingdong1221@163.com](mailto:bingdong1221@163.com)) 145 West Changle Road, Xi’an 710032, Shaanxi, China.

^†^ Na Zhao, Ying-Feng Gao and Lei Bao contributed equally to this work.


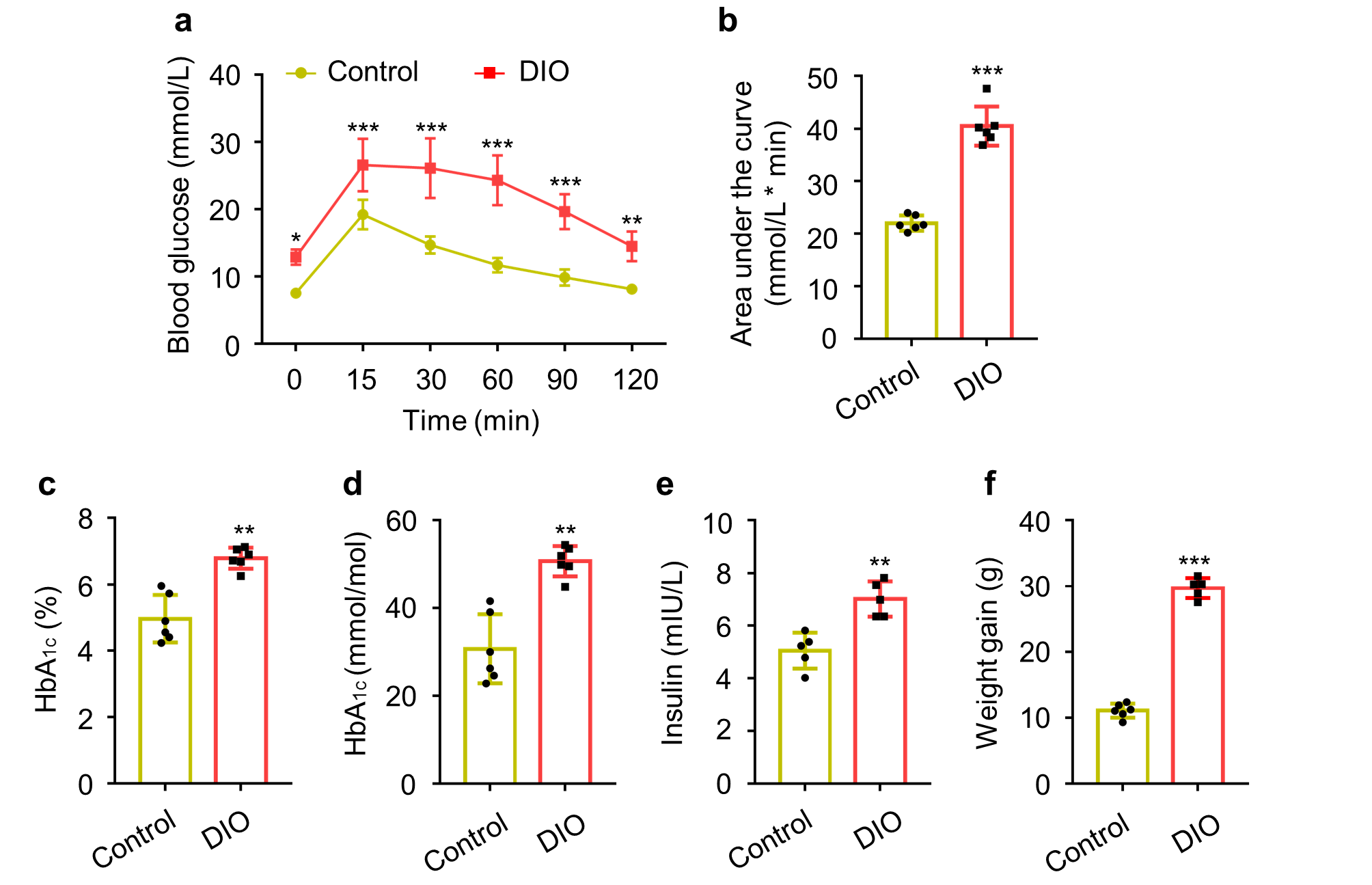


**Figure S1. Glucose homeostasis in control and DIO mice.** (a, b): Glucose tolerance was assessed by IPGTT. AUC above baseline was calculated as an index of glucose tolerance. (c-e): ELISA analyzed the levels of HbA_1c_, Hb and serum insulin. (f): weight gain after 16 weeks of HFD. The data are expressed as mean values ± SD. n=6 mice per group. *P < 0.05, **P < 0.01, ***P< 0.001.


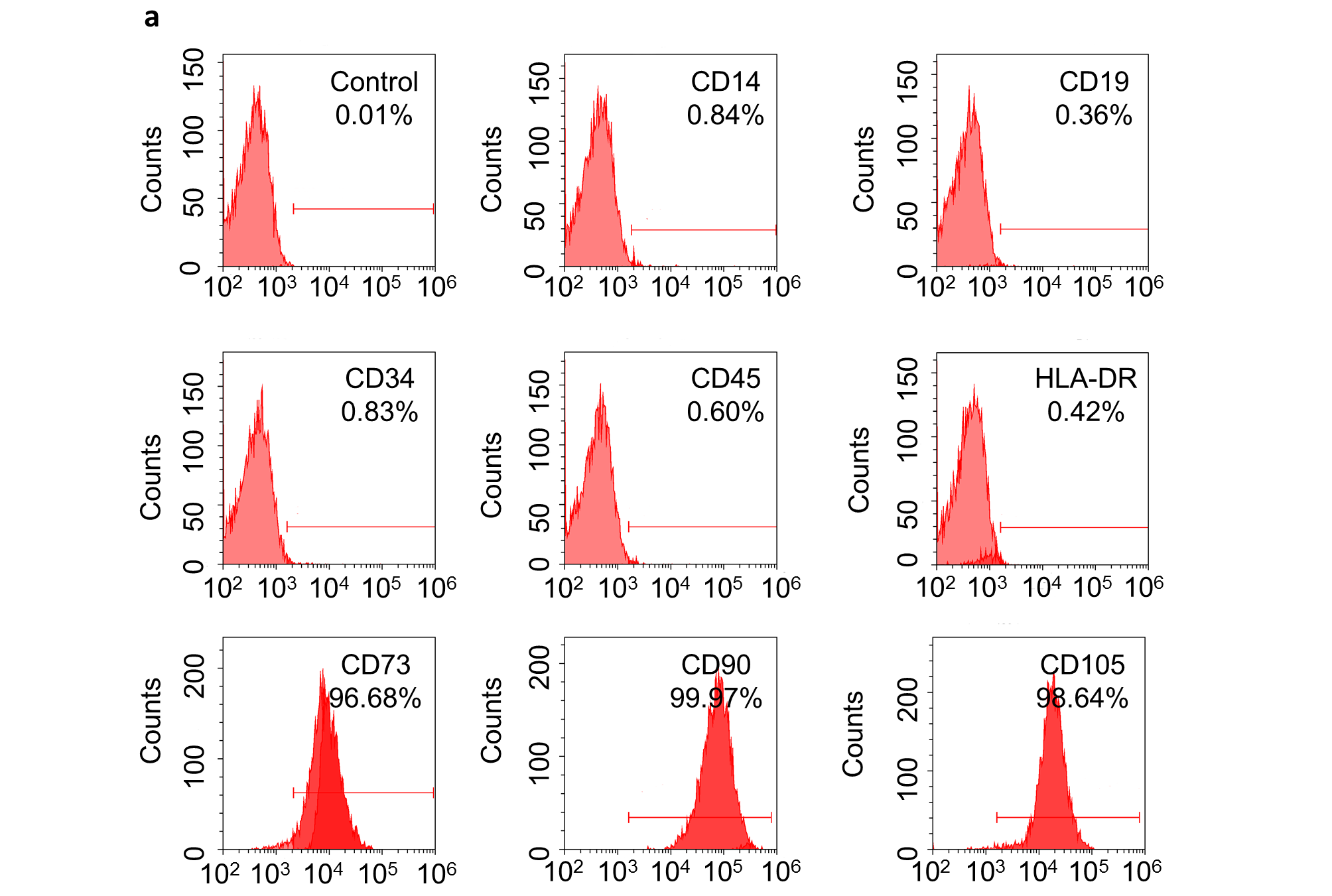


**Figure S2. The identification of UC-MSCs.** (a): Flow Cytometry results determining the UC-MSCs phenotype. UC-MSCs were stained with FITC-labeled CD14, CD19, CD73, HLA-DR and PE-labeled, CD34, CD45, CD90, CD105.


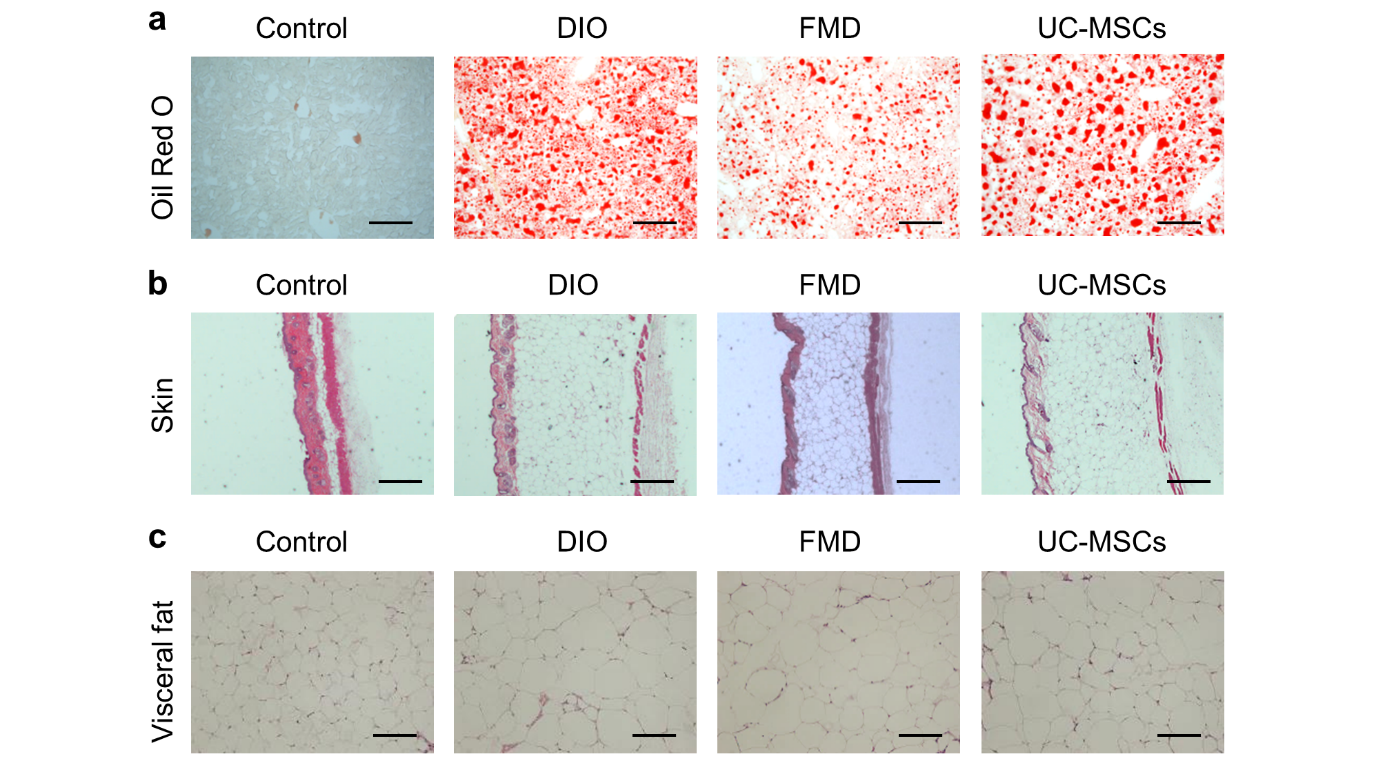


**Figure S3. Histomorphological changes of liver, skin and visceral fat.** (a): Liver steatosis were analyzed through staining with Oil Red O (Scale bar, 200 μm). (b): H&E staining of skin (Scale bar, 1 mm). (c): H&E staining of visceral fat (Scale bar, 100 μm).


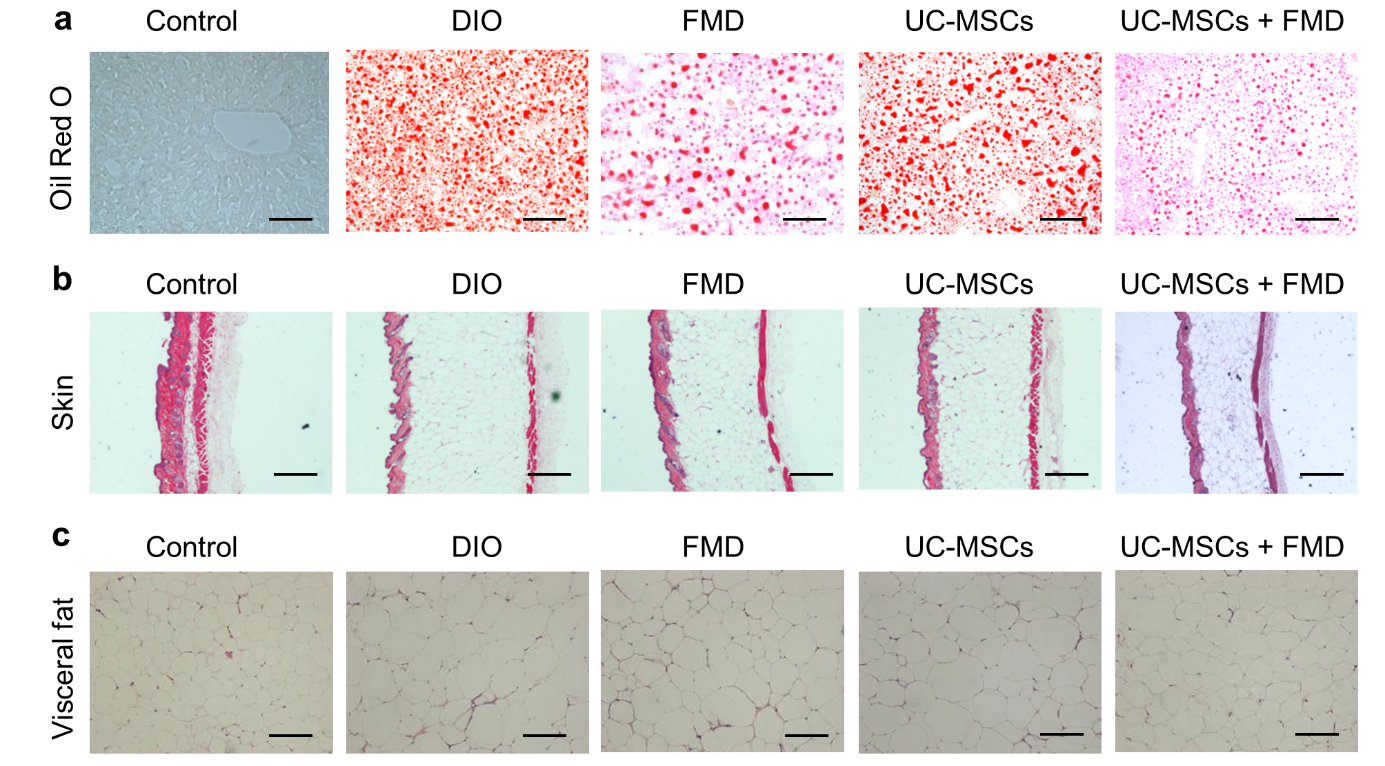


**Figure S4. Histomorphological changes of liver, skin and visceral fat.** (a): Liver steatosis were analyzed through staining with Oil Red O (Scale bar, 200 μm). (b): H&E staining of skin (Scale bar, 1 mm). (c): H&E staining of visceral fat (Scale bar, 100 μm).


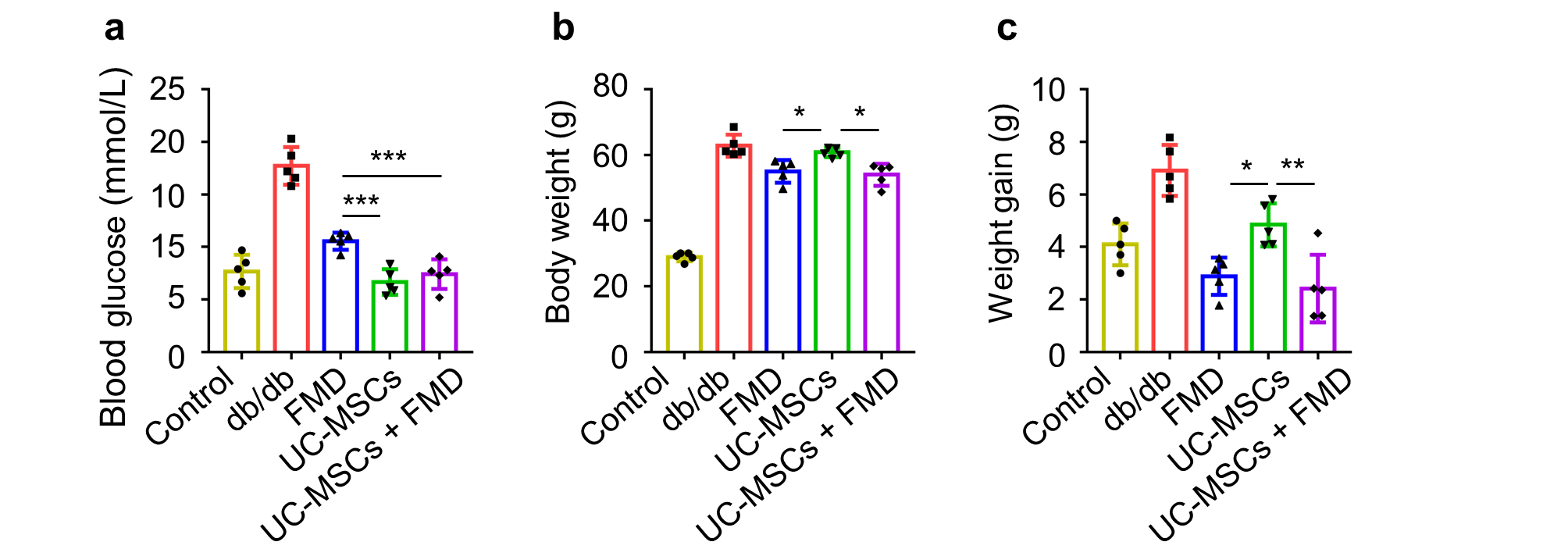


**Figure S5. The effect of UC-MSCs combined with FMD on blood glucose and body weight in db/db mice.** (a-c): Blood glucose and body weight were determined after fasting 6h at sacrificed. The data are expressed as mean values ± SD. n=6 mice per group. *P < 0.05, **P < 0.01, ***P< 0.001.
